# 

**Published:** 1906-10

**Authors:** 


VOL. LXII. OCTOBER, 1906. NO. 3

### BUFFALO MEDICAL JOURNAL

Established 1845 by AUSTIN FLINT, M. D.

INDEXED

OCT. 13 1906

EDITOR:

WILLIAM WARREN POTTER, M. D.

ASSISTANT EDITORS:

WILLIAM C. KRAUSS, M. D. NELSON W. WILSON, M. D.

ASSOCIATE EDITORS:

JAMES WRIGHT PUTNAM, M. D. ERNEST WENDE, M. D.
JOHN PARMENTER, M. D. JOHN A. MILLER, PH. D.
HARVEY R. GAYLORD, M. D. MAUD J. FRYE, M. D.
JOHN A. RAFTER, M. D.

